# Triangle of Care in a Crisis Resolution and Home Treatment Service: A CaseNote Audit From Telford & Wrekin

**DOI:** 10.1192/bjo.2026.11823

**Published:** 2026-06-30

**Authors:** Suleman Patel, AbdulAdl AbdulAdl Bolaji, Chandan Aldakatti, Aoife Aoife Turnbull, Theresa Mutasa

**Affiliations:** 1 MPFT T&W CRHT, Telford, United Kingdom; 2 Keele University, Newcastle-under-Lyme, United Kingdom

## Abstract

**Aims:**

The Triangle of Care (ToC) is a national framework, developed by the Carers Trust, comprising six standards to ensure carers are identified, supported and included in mental health care, which is especially pertinent for Crisis Resolution and Home Treatment (CRHT) “limited-contact” services.

Undertaking this Triangle of Care audit within CRHT is crucial because, in a short-term, high-intensity, limited-contact crisis setting, it checks whether carers are identified, supported and meaningfully involved (notably around consent and discharge planning), assures compliance with Trust policy and the Health and Care Act 2022 duty to involve carers, and drives a targeted action plan with scheduled re-audit to secure measurable improvements.

We hypothesised that compliance would be high for staff training and availability of carer information/support, but lower for documentation-dependent items such as consent and recording carers’ special circumstances.

**Methods:**

Retrospective review of 30 cases over 6–8 weeks (July–September 2025), excluding instances where no carer was identified despite reasonable enquiry.

Data sources included electronic case notes (carer identification, consent, documentation, discharge planning), staff training records, qualitylead feedback, and local policy/pack review.

A threecase pilot established interrater consistency; a subsample was checked by a senior colleague and carer lead for validation, in line with standard audit methodology.

**Results:**

Overall compliance across ToC standards was 77% (Green/Amber).

By individual standards:

- S1 Identification 55%
- S2 Staff training 100%
- S3 Confidentiality/information sharing 50%
- S4 Defined carer roles 67%
- S5 Carer introduction & information 90%
- S6 Range of carer support 100%.

Sub-items illustrating key gaps included:

- Recording special circumstances 27%
- Documented consent/limits 54%
- Routine discussions on sharing information 45%
- Revisiting non-disclosure 33%
- Offering support when disclosure declined 0%
- Carer involvement in discharge planning 50%

Strengths included universal carer-awareness training, an accessible introduction/information pack (including cultural/language considerations), and comprehensive signposting to support.

**Conclusion:**

Findings support the hypothesis: the CRHT team showed strong performance for staff training and information/support availability. There is much needed for improvement in consent processes, systematic recording of carer special circumstances, and consolidation of local carer-champion arrangements. These improvements would be an essential element in enhancing care for high-risk patients. Supporting wider organisational objectives to promote recovery in the community.

An action plan is in development to enhance service user and carer experience with a re-audit scheduled for 2026.

